# Synthetic lethal combination of CHK1 and WEE1 inhibition for treatment of castration-resistant prostate cancer

**DOI:** 10.21203/rs.3.rs-3564450/v1

**Published:** 2023-11-08

**Authors:** Qiming Wang, Yapeng Chao, Yuzhou Chen, Wenxiao Zheng, Kathryn Demanelis, Yu Liu, Jaclyn Connelly, Hong Wang

**Affiliations:** University of Pittsburgh School of Medicine; University of Pittsburgh Cancer Institute; University of Pittsburgh Cancer Institute; University of Pittsburgh Cancer Institute; University of Pittsburgh Cancer Institute; University of Pittsburgh Cancer Institute; University of Pittsburgh Cancer Institute; University of Pittsburgh Cancer Institute

**Keywords:** WEE1, CHK1, cell cycle, synthetic lethal, kinase inhibitor, prostate cancer

## Abstract

WEE1 and CHEK1 (CHK1) kinases are critical regulators of the G2/M cell cycle checkpoint and DNA damage response pathways. The WEE1 inhibitor AZD1775 and the CHK1 inhibitor SRA737 are in clinical trials for various cancers, but have not been examined in prostate cancer, particularly castration-resistant (CRPC) and neuroendocrine prostate cancers (NEPC). Our data demonstrated elevated WEE1 and CHK1 expressions in CRPC/NEPC cell lines and patient samples. AZD1775 resulted in rapid and potent cell killing with comparable IC50s across different prostate cancer cell lines, while SRA737 displayed time-dependent progressive cell killing with 10- to 20-fold differences in IC50s. Notably, their combination synergistically reduced the viability of all CRPC cell lines and tumor spheroids in a concentration- and time-dependent manner. Importantly, in a transgenic mouse model of NEPC, both agents alone or in combination suppressed tumor growth, improved overall survival, and reduced the incidence of distant metastases, with SRA737 exhibiting remarkable single agent anticancer activity. Mechanistically, SRA737 synergized with AZD1775 by blocking AZD1775-induced feedback activation of CHK1 in prostate cancer cells, resulting in increased mitotic entry and accumulation of DNA damage. In summary, this preclinical study shows that CHK1 inhibitor SRA737 alone and its combination with AZD1775 offer potential effective treatments for CRPC and NEPC.

## Introduction

Prostate cancer is the most common noncutaneous malignancy and the second leading cause of cancer-related deaths among men in the United States. Most prostate cancers are adenocarcinomas which are responsive to hormonal therapy, and the mainstay of treatment is medical castration by androgen deprivation therapy (ADT). Although it leads to favorable responses initially, almost all patients will inevitably relapse to a stage known as metastatic castration-resistant prostate cancer (mCRPC). Although mCRPC can still be treated with newer antiandrogens (abiraterone acetate and enzalutamide), their effectiveness is short-lived, and resistance quickly develops with a significant portion of recurrent CRPC belonging to treatment-related neuroendocrine prostate cancer (tNEPC), an aggressive lethal subtype of CRPC with limited treatment options and poor response to AR-targeted therapies. Novel treatments and effective combination strategies are urgently needed to improve the survival of patients with advanced-stage prostate cancer.

The core cell cycle machinery controlling the G2/M checkpoint is the CDK1/cyclin B complex. The activity of this complex is regulated by a group of protein kinases and phosphatases. Among them, the protein kinase WEE1, checkpoint kinase 1 (CHK1), and phosphatase CDC25C are central regulators of this complex. WEE1 inhibits CDC2/CDK1 activity by phosphorylating Tyr15 (Y15) of CDK1, which induces G2 arrest to allow cells to repair their DNA before entering mitosis. Additionally, WEE1 activity safeguards DNA replication in the S phase by limiting CDK2 activity, and inhibition of WEE1 increases replication stress and genomic instability [1]. CDC25C, conversely, catalyzes the dephosphorylation of Y15 on CDK1, which activates CDK1/cyclin B and enables mitotic entry after the completion of DNA repair. In contrast, CHK1 catalyzes the inhibitory phosphorylation of CDC25C, accumulating inactive Y15-phosphorylated CDK1, resulting in G2/M arrest. CHK1 is also responsible for the repair response to single-strand breaks and stalled replication forks in late S phase, leading to G2/M arrest [2]. In cancer cells, loss of the tumor suppressor gene *TP53* abrogates the G1 checkpoint, causing cancer cells to rely on the activation of the G2/M checkpoint to repair damaged DNA arising from replication stress or chemotherapy treatments [3]. Thus, compared to normal cells, these cancers could be particularly susceptible to G2/M checkpoint-targeted therapies, such as WEE1 or CHK1 inhibitors. Inhibition of WEE1 or CHK1 abrogates the G2/M checkpoint and forces cells to enter mitosis prematurely with unrepaired DNA, ultimately leading to mitotic catastrophe and apoptosis [4, 5].

WEE1 was reported to be differentially upregulated in NEPC tumors compared to androgen-sensitive prostate adenocarcinomas [6]. WEE1 expression is also actively associated with neuroendocrine activity and is negatively correlated with AR activity [6]. The first-in-class WEE1 inhibitor AZD1775 (adavosertib, MK-1775) has been shown to sensitize p53-deficient tumor cells to DNA-damaging chemotherapeutic agents *in vitro* and *in vivo* [7, 8] and is efficacious in multiple NE cancers including neuroblastoma [9], small cell lung cancer [10], and medulloblastoma [11]. Similarly, CHK1 inhibitors have been shown to synergize with various chemotherapies and ionizing radiation [12]. Increased genomic aberration in advanced cancer often makes tumors more susceptible to CHK1 inhibitors that target the DNA damage response (DDR) and G2/M checkpoint. Indeed, it has been shown that loss of both tumor suppressors *TP53* and *RB1* upregulates CHK1, resulting in tumor cells dependent on ATR/CHK1 signaling to activate the G2/M checkpoint to arrest cells for the repair of damaged DNA [13]. Although resistant to single-agent therapies, NEPC tumor cells with TP53/RB1 loss were highly responsive to the combination of PARP and ATR inhibition [14]. Despite the promising anticancer effects of AZD1775 and SRA737 in many cancers, their therapeutic value as single agents or in combination for prostate cancer has not been thoroughly investigated.

In this study, we explored the therapeutic potentials of WEE1 and CHK1 inhibition and identified an effective synthetic lethal WEE1 and CHK1 inhibitor combination as a potential treatment of CRPC and NEPC. Using an autochthonous castrated TRAMP mouse model, we showed that AZD1775 and SRA737 alone and in combination cooperatively suppressed tumor progression and metastasis and improved the overall survival of mice with NEPC tumors. The synergy of CHK1 and WEE1 inhibitors centered on cell death induced by abrogate of the G2/M and premature mitotic entry. Taken together, our study provided compelling preclinical evidence for translating WEE1 and CHK1 inhibitors and their combination for treatment of castration-resistant lethal prostate cancers.

## Materials and Methods

### Cell culture, reagents, and compounds

AZD1775, and SRA737 (5-[[4-[[(2R)-2-morpholinylmethyl]amino]-5-(trifluoromethyl)-2-pyridinyl]amino]-2-pyrazinecarbonitrile) were purchased from ChemieTek. DMSO was purchased from Sigma-Aldrich.

RWPE-1, LAPC4, LNCaP C4-2B, 22Rv1, VCaP, DU145, and NCI-H660 cell lines were obtained from the American Type Culture Collection (ATCC) and cultured under the vendor’s recommended conditions. Cells were grown in HITE medium (NCI-H660), Minimum Essential Medium (MEM) supplemented with Eagle’s salt and L-glutamine (DU145), Dulbecco’s MEM (VCaP), RPMI-1640 (LAPC4, LNCaP C4-2B, 22Rv1), or Ham’s F-12 (PC3-ML) medium supplemented with 10% FBS and 1× penicillin/streptomycin. Keratinocyte-SFM medium (ATCC) was used for RWPE-1. The cultures were maintained at 37°C in a humidified incubator containing 5% CO_2_.

Antibodies against p-S642-WEE1, Cleaved PARP, p-S345-CHK1, CHK1, p-Y15-cdc2, cdc2, p-S216-CDC25C, CDC25C, p-S139-His H2A.X, p-S10-HistoneH3, Cleaved Caspase-3 were purchased from Cell Signaling Technology. WEE1 was from Abcam. GAPDH was purchased from Santa Cruz Biotechnology. Goat anti-rabbit and anti-mouse secondary antibodies were purchased from Bio-Rad.

### Bioinformatic analysis

The clinical information data of prostate adenocarcinoma from the Cancer Genome Atlas (TCGA-PRAD) were acquired (untill May 26, 2023), duplicates and samples with missing information were removed (https://portal.gdc.cancer.gov/). The distribution of pathological extension of the tumor (T staging) and spread to the lymph nodes (N staging) in the samples were compared between samples in the first and fourth quartiles of *CHK1* (or *WEE1*) expression levels. M staging was excluded from the analysis due to the need for more information on metastasis in most samples. Chi-square tests and ggplot2 (v3.3.6) were performed using the R (v4.2.1) for statistical analyses.

A different dataset from the SU2C/PCF Dream Team [15] was used to explore the potential relationship between *CHK1* and *WEE1* and the survival status of patients with metastatic castration-resistant prostate cancer (mCRPC). Samples with complete information on outcome events and overall survival were maintained. Kaplan-Meier curves were used to visualize the survival status of those expressing higher *CHK1* (or *WEE1*) versus those expressing lower than the median. Calculation and visualization of the time-dependent area under the curve (AUC) were achieved using timeROC (v0.4), and ggplot2 (v3.3.6) in R (v4.2.1), according to the hypothesis developed by Heagerty *et al.*[16]. For statistical analyses, log-rank (Mantel-Cox) tests were applied using GraphPad Prism 9.0; *p* less than 0.05 was considered statistically significant.

### Incucyte Cell proliferation assay and 3D spheroid assay

Prostate cancer cell lines (C4-2B, DU145, PC3, 22Rv1, and LASCPC-01) were infected with NucLight Red (NR) Lentivirus reagent (Sartorius, Bohemia, NY) to establish stable cell lines expressing the red fluorescent protein mKate2. NR stable cells were seeded at 6,000–9,000 cells/well in 96-well plates in growth medium containing 10% FBS. After applying the inhibitors, the plates were placed in the IncuCyte S3 Live Cell Imaging System (Sartorius), where real-time images were captured every 6 h for 4–7 days at ×10 or ×20 magnification throughout the experiment. The red fluorescent signal of the cells was analyzed using the IncuCyte cell-by-cell module (Sartorius).

For the 3D spheroid assay, LASCPC-01 cells were seeded on ultra-low attachment plates and centrifuged at 300 g for 3 min. Cell growth and shape were estimated using a spheroid module on an IncuCyte S3 (Sartorius). IC50 was determined using Prism, and synergy scores were obtained using SynergyFinder software [17].

### Cell viability assay

For cell lines (LNCaP and H660) that were not suitable for IncuCyte analysis because of their inability to establish NR-stable cells or cells growing in clusters, cell viability was measured by Cell Counting Kit-8 (MedChemExpress) according to the manufacturer’s protocol. Briefly, the cells were plated at a density of ~ 7000 cells/well in 96-well plates. After treating the cells with different doses of inhibitors for 72 hr, CCK-8 reagent containing a highly water-soluble tetrazolium salt WST-8 [2-(2- methoxy-4-nitrophenyl)-3-(4- nitrophenyl)-5-(2,4-disulfophenyl)-2H-tetrazolium, monosodium salt] was added to the cells in each well, followed by incubation for 2–3 h. Cell proliferation and viability were determined by measuring the OD at 450 nm using a Synergy H1 hybrid reader (BioTek). The percentage of control cells was calculated as a measure of the cell viability.

### TRAMP mice

All animal experiments were conducted in accordance with the guidelines of the University of Pittsburgh Institutional Animal Care and Use Committee guidelines. B6 TRAMP mice (C57BL/6-Tg (TRAMP) 8247Ng/J, stock# 003135) heterozygous for the probasin-driven SV40 T antigen were obtained from the Jackson Laboratory (Bar Harbor, ME). Homozygous female B6 TRAMP mice were generated by intercrossing heterozygous B6 TRAMP mice. The mouse colony was maintained and genotyped by PCR using tail DNA as previously described [18]. Heterozygous [TRAMP × FVB] F1 mice were generated by crossbreeding homozygous female B6 TRAMP mice with wild-type FVB male mice. Male [TRAMP × FVB] F1 mice were castrated at 16–17 weeks of age. Drug treatment was initiated at 20–21 weeks of various lengths. Mice were sacrificed at 24–25 weeks. In mice with palpable internal (prostate) tumors, tumor volume was measured every two days, and body weight was recorded every four days. For the survival study, mice with palpable tumors were selected for treatment. Mice were monitored for tumor size and body weight until deceased or moribund, when mice were dissected and counted as dead.

### Mouse castration

TRAMP mice were anesthetized by isoflurane inhalation. Testes were gently pushed gently into the scrotum. A small incision (0.5 cm) was made along the midline of the scrotum under sterile conditions. Both testes were surgically removed. The spermatic cord and vascular plexus were tied using sterile sutures to prevent bleeding. The skin incision was closed using a wound clip, which was removed 7–10 days after the surgery.

### Mouse tissue collection

At necropsy, the urogenital tract (UGT), consisting of the prostate (including dorsolateral, ventral, and anterior lobes), urethra, bladder, and seminal vesicle was carefully dissected using RNA-free surgical scissors. The bladder was deflated to release urine. The UGT was weighed, and the UGT/body weight ratio was calculated as UGT ÷ body weight × 100.

Tumors were further separated from the UGT under a dissection microscope (4×). For histopathology, one set of prostate lobes and tumors was fixed in 10% neutral buffered formalin for paraffin embedding, sectioning, and immunohistochemistry (IHC) analysis. The remaining lobes or tumors were flash-frozen for western blot analysis.

### Western blotting analysis

The cells were lysed in IP lysis buffer (50 mM Tris-HCl pH 7.5, 150 mM NaCl, 1.5 mM MgCl_2_, 10% glycerol, 1% Triton X-100, 5 mM EGTA, 1 mM Na_3_VO_4_, 10 mM NaF, 1 mM β-glycerophosphate and protease inhibitor cocktail). Western blotting was performed as described previously [19]. Briefly, trypsinized or suspended cells were washed and collected by centrifugation in a lysis buffer. After brief sonication, the proteins were quantified by the BCA method (Pierce). 25 μg of protein were resolved in 10% - 12% SDS-PAGE. Proteins were transferred to nitrocellulose membranes and blocked in 5% milk-TBST for 1 hr at room temperature. Primary and secondary antibodies were diluted in 5% BSA-TBST and the blocking buffer, respectively. The immunoblots were incubated with primary antibody at 4°C overnight, followed by blotting with secondary antibodies for 1 hr at room temperature. ECL chemiluminescence (Pierce) was used to develop blots.

### Immunofluorescence (IF) staining and microscopy

IF staining was performed as previously described [19]. Briefly, cells grown on poly-D-lysine-coated coverslips were washed and fixed in 4% paraformaldehyde at room temperature, followed by washing and blocking in a buffer containing 5% normal goat serum and 0.3% Triton X-100 for 1 hr at room temperature. The cells were incubated with primary antibodies overnight at 4°C, followed by incubation with secondary antibodies for 1 hr at room temperature. The cells were counterstained with DAPI (1 μg/ml), and coverslips were mounted using ProLong Gold antifade reagent (Invitrogen). The cells were analyzed using an Olympus Fluoview (FV1000) confocal microscope at 60 ×/1.45 objectives.

### siRNA Transfection

WEE1 siRNAs were obtained from Integrated DNA Technologies (Coralville). Cells were transfected with siRNAs using Lipofectamine RNAiMAX Transfection Reagent (Invitrogen), according to the manufacturer’s instructions.

### Statistical analysis

Statistical analyses for cellular data were performed using GraphPad Prism 9.0 software (GraphPad Software, Inc., La Jolla, CA, USA). Continuous outcomes were analyzed using the Student’s *t*-test for comparison between two groups (two-tailed) or ANOVA with Tukey’s post-hoc analysis for three or more groups. The correlation of variables was studied using linear regression analysis. Replicate experiments were analyzed both independently and jointly by controlling for batch effects. All values are represented as the Mean ± Standard Error of the Mean (SEM).

For all animal experiments, the mice were randomized into treatment groups. The mouse group allocation was blinded at all stages of the experiment and analysis. All the mice and data points from each experimental group were included in the analysis. Statistical analyses of mouse data were performed using R (v4.1.2), and the packages used were *lmerTest* and *emmeans*. Data from each experiment were analyzed separately. Tumors in mice were separated into two types (palpable [T] and unpalpable [UGT]). The tumor weight and volume were studied within the tumor or UGT strata. They were also studied in all the data with linear mixed effects models, where tumor type (T vs. UGT) was one of the fixed effects. A Box-Cox transformation was applied if the data were not normally distributed. The survival function in each vehicle or treatment group was estimated using the Kaplan-Meier estimator, and differences in survival were assessed using the log-rank test. Associations between body weight and tumor volume and treatment were examined using linear mixed models (using the *lmer* function in R), where the following model was applied: body weight (or tumor volume) ~ day of measurement + treatment*day of measurement + (1|eartag) + (day of measurement|eartag), where both a random slope and a random intercept term were included. Pairwise contrasts were performed (using *emtrends* and the *contrast* function in R), where degrees of freedom were estimated using the Kenward-Roger method, and p-values were adjusted using the Tukey method. Fisher’s exact test was used to compare the distribution of metastases and recurrences among the groups. ANOVA was used to compare final body weight and tumor weight in all animal experiments, and p-values were obtained from the corresponding F-tests. For all analyses, a *p*-value < 0.05 was considered statistically significant (* *p* < 0.05, ** *p* < 0.01, *** *p* < 0.001, **** *p* < 0.0001, ns: not significant).

## Results

### Elevated expression of WEE1 and CHK1 in CRPC and NEPC cells and tumor samples.

**Figure 1:**
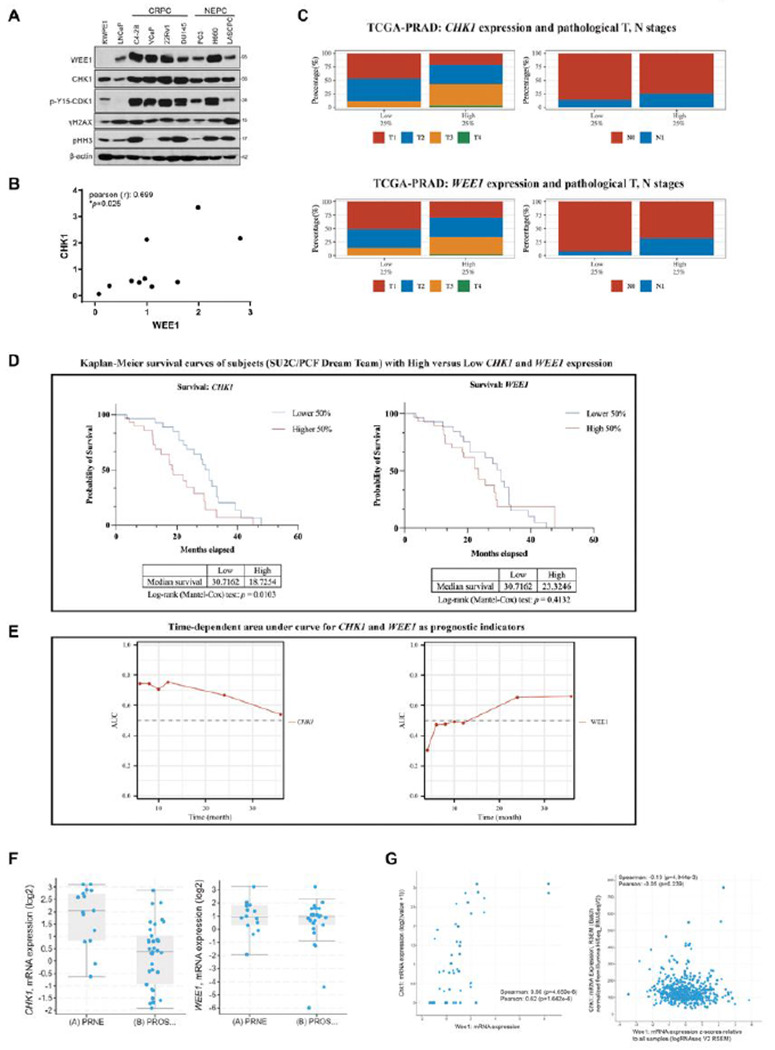
Expressions of WEE1 and CHK1 and effects of WEE1 inhibition or depletion in CRPC cell lines. **A.** WB analysis on a panel of prostate cancer cells. Cells in the log phase were collected for WB analysis. **B.** Correlation of WEE1 and CHK1 expression in prostate cancer cells. Scatter plot of WEE1 and CHK1 WB quantification. Each point is the average from three independent experiments. **C.** The distribution of samples at T and N stages in the first and fourth quartile of *CHK1* (*top*) or *WEE1* (*bottom*) expression levels. The abscissa represents different groups, and the ordinate represents the percentage of clinical sample information in the corresponding group. Different colors represent different pathological stages. **D.** Kaplan-Meier curves showing survival status in subjects with high versus low expression levels of *CHK1* and *WEE1*. **E.** Time-dependent area under the curve when using *CHK1* and *WEE1* as prognostic indicators. **F.** *CHK1* and *WEE1* mRNA levels in NEPC (PRNE) and prostate adenocarcinoma (PROS) (*p* = 7.299E-4 for *CHK1;* *p*= 0.398 for *WEE1*). **G.** Scatter plot showing *CHK1* mRNA correlated to *WEE1* mRNA expression in NEPC (TCGA, NEPC, Nat Med 2016 [20]), but not in prostate adenocarcinoma (TCGA, PanCancer Atlas).

To assess the potential therapeutic value of targeting WEE1 and CHK1 in prostate cancer, we first evaluated WEE1 and CHK1 expression in a panel of prostate cancer cell lines with different genetic backgrounds. As shown in Fig. 1A, the CRPC cell lines (C4-2B, VCaP 22Rv1, and DU145) exhibited enhanced WEE1 and CHK1 expression compared to those in the androgen-sensitive cell lines LNCaP and LAPC4, and the immortalized normal human prostate epithelial cell line RWPE1 (LAPC4 depicted in **Supplemental Fig. S1A**). The elevated expression of WEE1 and CHK1 in LNCaP-derived C4-2B CRPC cells relative to parental LNCaP cells indicates that the upregulation of these two genes may contribute to castration resistance. The expression of WEE1 and CHK1 was also elevated in NCI-H660 (H660), an authentic NEPC cell line derived from a clinical NEPC patient tumor. Their expression in two other NEPC cell lines, PC3 and LASCPC-01 (an NEPC cell line derived from benign human prostate tissue through transformation with the MYCN and myristoylated AKT1 oncogenes), was similar to that of LNCaP cells, but higher than that in normal RWPE1 cells. WEE1 protein expression in these cells significantly correlated with that of CHK1 (Pearson’s r = 0.70, *p* = 0.02, n = 3 Fig. 1B) and p-Y15-CDK1 (**Fig. S1B**), an indicator of the activated G2/M checkpoint, suggesting that these CRPC and NEPC cells may rely on WEE1- and CHK1-mediated G2/M control for survival, and thereby could be sensitive to agents targeting this pathway.

To assess the significance of *WEE1* and *CHK1* gene expression in human prostate cancer patients, the relationship between *CHK1* and *WEE1* mRNA and the pathological features and survival of patients with prostate adenocarcinoma was analyzed using data from The Cancer Genome Atlas (TCGA-PRAD). As shown in Fig. 1C, significantly higher proportions of samples at more advanced stages were observed in the upper quartile than in the lower quartile of *CHK1* expression (*p* = 10E-7.26 for T staging, *p* = 0.039 for N staging, **Supplemental Table S1**). Similar results were observed between the *WEE1* expression level and pathological T&N staging (*p* = 10E-3.41 for T staging, *p* = 10E-4.16 for N staging, **Supplemental Table S1**). Analysis of overall survival in patients with high or low *CHK1* expression indicated that patients with *CHK1* expression levels lower than the median achieved a significantly higher probability of survival (*p* = 0.0103) with a median survival time of approximately 31 months, compared to those expressing *CHK1* higher than the median (Fig. 1D). A similar trend was observed between *WEE1* expression and survival status, although the difference was not statistically significant (*p* = 0.4132) (Fig. 1D). The prognostic values of *CHK1* and *WEE1* were further explored as the survival status became more time-dependent as the disease progressed, especially among subjects with mCRPC. In line with the significant prognostic value of *CHK1* as suggested by the Kaplan-Meier (K-M) curves, its power as a prognostic indicator was confirmed by the time-dependent AUC throughout the first 24 months (AUC above 0.6, Fig. 1E). On the other hand, the low AUC values of *WEE1* in the first 12 months also matched the insignificant prognostic value reflected by the K-M curves. However, the AUC value of *WEE1* went above 0.6 after two years, suggesting an increased importance of *WEE1* in contrast to *CHK1* at later stages of disease progression (Fig. 1E, **Supplemental Table S2**). Interestingly, compared to prostate adenocarcinoma, *CHK1* mRNA was markedly upregulated in NEPC tumors (*p* = 7.299E-4), while that of *WEE1* was not (*p* = 0.398) (Fig. 1F)., The expression of *CHK1* mRNA was positively correlated with the expression of *WEE1* mRNA in NEPC (Spearman’s r = 0.6, *p* = 4.65E-6), but not in prostate adenocarcinoma (Spearman’s r = −0.13, *p* = 4.944E-3) (Fig. 1G). Overall, these data provide a rationale for targeting *WEE1* and *CHK1* in advanced stages of prostate cancer, with *CHK1* being more strongly indicated.

### Effects of WEE1 or CHK1 inhibition on the survival of CRPC and NEPC cells.

**Figure 2:**
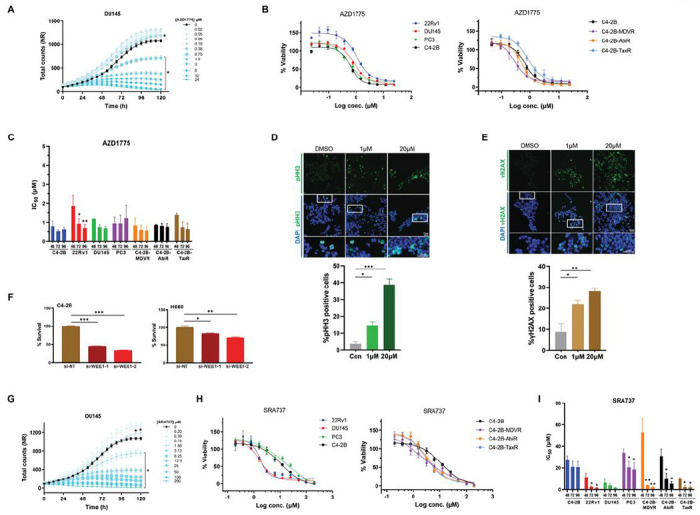
Effects of AZD1775 and SRA737 alone on the viability of CRPC and NEPC prostate cancer cells. **A.** Time-course of DU145-NR cell viability treated with or without increasing concentrations of AZD1775. Total cell counts were measured by IncuCyte live-cell imaging. Data from one of three representative experiments are shown, *, p < 0.05 comparing 0 to all doses, two-way ANOVA. **B.** Dose-response curves of prostate cancer cell lines treated with AZD1775 for 96 h are shown. Data from one of three representative experiments with quadruplicate determinations. There is no significance in comparing any two cell lines (*p* > 0.05, two-way ANOVA). **C.** IC50 values of AZD1775 in different cell lines. There is no significance comparing 48 h to 72 or 96 h within each cell line except 22Rv1. **D.** IF staining of mitotic cells using pHH3 antibody (green) in H660 cells treated with or without AZD1775. **E.** Images and quantification of DNA damage by staining for γH2AX-positive (green) H660 cells before and afterAZD1775 treatment. Nuclei were stained by DAPI. Representative images are shown in E and F. **F.** Effects of WEE1 knockdown by siRNAs on survival of H660 and C4-2B cells. Cell viability was determined by the CCK-8 assay. **G.** Time-course of DU145-NR proliferation treated with or without SRA737. *, *p* < 0.05 comparing 0 to doses ≥ 1.56 μM, no significance for doses ≤ 0.78 μM, two-way ANOVA. **H.** Dose-response curves of prostate cancer cell lines treated with SRA737 for 96 h are shown. ***, *p* < 0.001 comparing PC3 or C4-2B to DU145 or 22Rv1 (left); *, *p* < 0.05 comparing C4-2B with each resistant line (*right*), two-way ANOVA. **I.** IC50 values of SRA737 in different cell lines. 48 h was compared to 72 or 96 h within each cell line by t-test. Data in “D”-“F” are Mean ± SEM from three independent experiments. *, *p* < 0.05; **, *p* < 0.01, ***, *p* < 0.001.

Increased WEE1 and CHK1 expression in CRPC and NEPC cells suggests that these cells may depend on the G2/M checkpoint for survival, and may be sensitive to the inhibition of these two kinases. The WEE1 inhibitor AZD1775 and the CHK1 inhibitor SRA737 were selected to examine this possibility. Both inhibitors are in phase I/II clinical trials for various cancers [21–23]. AZD1775 has been evaluated in clinical trials for solid tumors, including mCRPC, but not in trials designed for prostate cancer. SRA737, on the other hand, has not been evaluated in the context of prostate cancer. This study examined the effects of AZD1775 and SRA737 on the proliferation and viability of CRPC and NEPC cells stably expressing the red fluorescent marker NucLight Red (NR) by real-time live-cell imaging. As shown in Fig. 2A, AZD1775 induced time- and concentration-dependent biphasic responses in DU145 cells, where at lower concentrations (≤ 0.38 μM for DU145), cell proliferation was stimulated as a consequence of increased mitosis due to inhibition of WEE1 while at higher concentrations (> 0.38 μM for DU145) decreased cell viability/proliferation was observed. Similar effects were observed in all other cell lines examined, including 22Rv1, PC3, and C4-2B, and three drug-resistant lines derived from C4-2B, C4-2B-MDVR (enzalutamide-resistant), C4-2B-TaxR (docetaxel-resistant), and C4-2B-AbiR (abiraterone-resistant) [24–26] (**Supplemental Fig. S2A**). The dose-response curves for AZD1775 at 96 h were plotted, showing a similar pattern of responses with IC50 values between 0.5-1 μM for all cell lines examined except for PC3, which was 2-fold higher (Fig. 2B and Table 1), and the responses did not correlate to WEE1 expression in these cells. The effect of AZD1775 was rapid and plateaued at 48 h and the IC50 values remained constant at 48, 72, 96 h in most cell lines (Fig. 2C). AZD1775 dose-dependently increased mitotic cell numbers, as measured by p-S10-Histone H3 (pHH3) immunofluorescence (IF) staining (Fig. 2D), and resulted in elevated DNA double-strand breaks, as measured by IF staining of p-S139-His H2A.X (γH2AX) foci (Fig. 2E). Accordingly, knockdown of WEE1 by siRNAs significantly reduced cell viability in C4-2B and H660 cells as compared to the control cells transfected with non-targeting siRNA (si-NT) (Fig. 2F). The analysis of SRA737 demonstrated a similar biphasic response in proliferation/viability of all cell lines examined, namely stimulation of proliferation at lower concentrations (≤ 0.78 μM for DU145) while inhibiting at higher concentrations (> 0.78 μM for DU145) (Fig. 2G and **supplemental Fig. S2B**). However, when dose-response curves for SRA737 were plotted at 96 h, apparent differences in sensitivity to SRA737 were observed between the cell lines. Specifically, 22Rv1 and DU145 were 10- to 20-fold more sensitive to SRA737 than were C4-2B, PC3, and H660 (Fig. 2H and Table 1). Similar to AZD1775, differences in sensitivity to SRA737 did not consistently correlate with CHK1 expression in these cells. Interestingly, the C4-2B drug-resistant cell lines also exhibited approximately 2 to 10-fold greater sensitivity to SRA737 than the parental C4-2B cells (Fig. 2H and Table 1), implying a potential value of SRA737 for therapy-resistant prostate cancer. Additionally, in contrast to AZD1775, SRA737 was more slow-acting, as a time-dependent decrease in IC50 values was observed in all sensitive cell lines. However, this was less apparent in the more resistant C4-2B and PC3 cell lines. A longer incubation time is required to achieve a maximal response to SRA737 (minimally 72 h) (Fig. 2I). Collectively, these data indicate that AZD1775 and SRA737 are effective therapeutic agents for CRPC and NEPC *in vitro*.

### Synthetic lethal effects of combined WEE1 and CHK1 inhibition in vitro.

**Figure 3:**
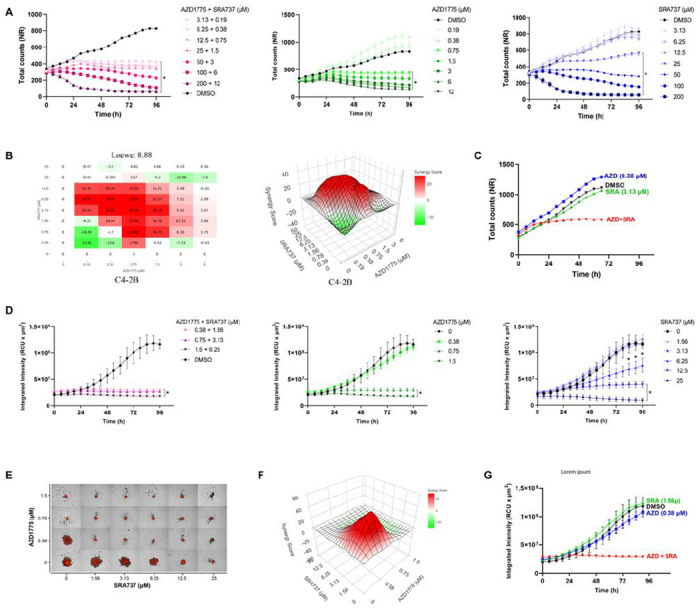
Synthetic lethality of WEE1 and CHK1 inhibition *in vitro*. **A.** Time-course of C4-2B-NR cell viability treated with AZD1775 and SRA737 alone and in combination for 4 days. Total cell counts were measured by IncuCyte live-cell imaging. Data from one of three representative experiments are shown. Significant doses compared to 0 were marked by * (*p* < 0.05, two-way ANOVA). **B.** Cooperativity assessment of AZD1775 and SRA737 in C4-2B cells. Drug interactions at day 4 were analyzed by SyngeryFinder Plus. Representative Loewe cooperativity heatmap and 3D plots shown are shown. **C.** Synergistic effects of combined low doses of AZD1775 and SRA737 over time compared to each drug alone. Data are extracted from the cooperativity screen in “B”. **D.** The growth kinetics of LASCPC-NR tumor spheroids treated with AZD1775 and SRA737 alone and combined for 4 days. The integrated intensity of spheroids was measured using a spheroid analysis module in IncuCyte. Error bars are SEM (n = 3) from representative one of three independent experiments. Significant doses compared to 0 were marked by * (*p* < 0.05, two-way ANOVA and t-test). **E.** Fluorescence images of LASCPC-NR tumor spheroids after 4 days of combined AZD1775 and SRA737 treatment. Representative images from one of three experiments are shown. **F.** 3D Loewe plot of AZD1775 and SRA737 cooperativity assessment at 96 h is shown. **G.** Quantitative measure of tumor spheroid growth kinetics over time treated with AZD1775 and SRA737 alone or in combination at low doses. Data are the Mean ± SEM from one of three representative experiments with quadruplicate determinations. *, *p* < 0.05 comparing DMSO to the combination; not significantly comparing DMSO to SRA or AZD alone.

The fact that WEE1 and CHK1 act through different pathways to modulate the CDK1/cyclin B complex implies that their inhibition can be combined to enhance abrogation of the G2/M checkpoint. To test this, we examined the antiproliferative effects of AZD1775 and SRA737 alone and in combination in CRPC and NEPC cells using live-cell imaging. As shown in Fig. 3A, the combination of AZD1775 and SRA737 resulted in more significant inhibition of cell proliferation/viability as compared to each drug alone, and the effect was most apparent at non-toxic, low doses of AZD1775 (0.19–0.38 μM) and SRA737 (3.13–6.25 μM) for C4-2B cells. A cooperativity screen was then conducted to further evaluate the interactions between the two drugs. As illustrated in Fig. 3B–C, strong synergy between the two drugs was demonstrated, as indicated by synergy scores obtained from the four synergy analysis models (Fig. 3B, Table 2). The most significant synergy was exemplified in a growth kinetic plot showing the combination of two non-toxic, low doses of AZD1775 (0.38 μM) and SRA737 (3.13 μM) resulted in nearly complete inhibition of C4-2B proliferation (Fig. 3C). Drug interaction analysis demonstrated similar synergistic effects between AZD1775 and SRA737 in 22Rv1, DU145, and PC3 cells (**Supplemental Fig. S3A-C** and Table 2), with PC3 NEPC cells displaying the greatest synergy score (**supplemental Fig. S3C**).

Because the NEPC cell line LASCPC grew in partially suspended clusters, NR-positive tumor spheroids were established for these cells. As shown in Fig. 3D, AZD1775 and SRA737 suppressed the growth of LASCPC tumor spheroids in time- and dose-dependent manners. Their combination resulted in greater retardation of growth kinetics than each drug alone. The cooperativity screen of the AZD1775 and SRA737 combination demonstrated strong synergy, as indicated by their synergistic 3D plot and scores (Fig. 3E–G, Table 2). In a representative spheroid growth plot, the combination of two non-toxic, low doses of AZD1775 (0.38 μM) and SRA737 (1.56 μM) completely eradicated the growth of LASCPC tumor spheroid. Collectively, these data demonstrate a strong synergy of AZD1775 and SRA737 combination in 2D CRPC and NEPC cell cultures and 3D tumor spheroids, suggesting that the combined inhibition of WEE1 and CHK1 may be an effective treatment for advanced prostate cancer.

### CHK1 inhibition synergized with WEE1 inhibition by blocking WEE1 inhibitor-induced feedback activation of CHK1 in prostate cancer cells.

**Figure 4:**
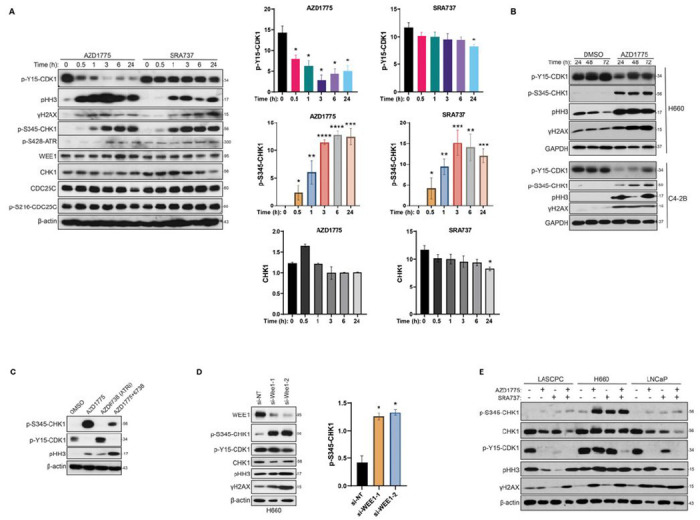
SRA737 synergized with AZD1775 by blocking AZD1775-induced CHK1 activation in prostate cancer cells. **A.** Differential effects of AZD1775 and SRA737 at molecular levels. DU145 cells were treated with AZD1775 (1 μM) and SRA737 (2 μM) for the indicated time, followed by Western blotting analysis (*left*). Levels of p-Y15-CDK1, p-S345-CHK1, and CHK2 were quantified by densitometry analysis using ImageJ (*right*). **B.** AZD1775 induced CHK1 activation in H660 and C4-2B cells. Western blotting analysis of H660 and C4-2B treated with control (DMSO) or AZD1775 for the indicated times. **C.** Inhibition of ATR attenuated AZD1775-induced CHK1 activation. DU145 cells were treated with AZD1775, AZD6738, and their combination for 3 h, followed by Western blotting analysis. **D.** Knockdown of WEE1-induced CHK1 activation. H660 cells were transfected to two WEE1 siRNAs for 5 days, followed by immunoblotting for indicated proteins (*left*). Quantification of p-S345-CHK1 in the Western blot was measured by densitometry analysis (*right*). **E.** AZD1775 and SRA737 synergized to inhibit p-Y15-CDK1 in prostate cancer cells. LASCPC, LNCaP, and H660 cells were treated with IC50 doses of AZD1775 (0.5 μM for H660 and LASCPC, 2 μM for LNCaP) and SRA737 (20 μM for H660, 8 μM for LASCPC, 1.5 μM for LNCaP) for twice of their doubling times. The cells were analyzed by Western blotting. All experiments were repeated at least three times, and representative images were shown.

In this study, we examined the molecular mechanisms underlying the synergistic effects of AZD1775 and SRA737 on prostate cancer cells. As shown in Fig. 4A, the two drugs at their respective IC50 concentrations had distinct effects on cell cycle and DNA damage markers in DU145 cells. AZD1775 rapidly inhibited p-Y15-CDK1 observed 30 min after treatment initiation, while SRA737 did not affect p-Y15-CDK1 for up to 24 h. Correspondingly, AZD1775 rapidly and potently stimulated mitotic entry (pHH3), which peaked at 3 h, while only a small effect was observed for SRA737. In contrast, both drugs induced time-dependent DNA damage (γH2AX) which peaked at 3 h for AZD1775 and 24 h for SRA737. Interestingly, SRA737 also caused a time-dependent downregulation of CHK1, which was visible after 3 h and progressed over time. Little effect was observed for p-S216-CDC25 or CDC25C at any time point, except at 24 h. These data imply that AZD1775 acts primarily by regulating the cell cycle, whereas SRA737 is a weaker cell cycle regulator and has more complex mechanisms of action, likely involving downregulation of CHK1 and induction of DNA damage. Moreover, both AZD1775 and SRA737 induced p-S345-CHK1 expression, an indicator of CHK1 activation by ATR (Fig. 4A). CHK1 inhibitors are known to induce p-S345-CHK1 through a CHK1 activity-dependent phosphatase cycle, where inhibition of CHK1 causes the accumulation of p-S345-CHK1 [27]. Inhibition of WEE1 by AZD1775 has also been shown to induce p-S345-CHK1 by activating ATR in other cancer cells [28], which may circumvent the effectiveness of WEE1 inhibition. AZD1775-induced p-S345-CHK1 was also observed in other prostate cancer cells, such as H660 and C4-2B, where increased p-S345-CHK1 was accompanied by increased pHH3 and γH3AX (Fig. 4B). This effect was dependent on ATR, because blocking ATR by AZD6738, an ATR inhibitor, significantly attenuated the levels of p-S345-CHK1 induced by AZD1775 and increased mitotic entry (pHH3) in DU145 cells (Fig. 4C). This was an on-target effect of WEE1 inhibition since knockdown of WEE1 also caused a robust increase in p-S345-CHK1, accompanied by increased mitosis (pHH3) and DNA damage (γH3AX) in H660 cells (Fig. 4D). Thus, these data provide a rationale for combining WEE1 inhibition with CHK1 inhibition to achieve synergistic killing of prostate cancer cells.

Our data demonstrated a strong synergy between AZD1775 and SRA737 in all prostate cancer lines regardless of their differential sensitivity to SRA737. To understand the molecular underpinnings of this observation, the effects of both agents on key cell cycle regulators were compared between SRA737-sensitive and -resistant cells. As shown in Fig. 4E, in SRA737-resistant H660 cells, both AZD1775 and SRA737 at their respective IC50 doses induced a robust increase in p-S345-CHK1, possibly contributing to the lack of effect on p-Y15-CDK1. This contrasted with the almost complete blockade of p-Y15-CDK1 in the two SRA737-sensitive LASCPC and LNCaP cell lines. Interestingly, despite the different responses to single drugs. The combination of AZD1775 and SRA737 was equally potent at inhibiting p-Y15-CDK1 in both sensitive and resistant cells, correlating well with the uniformly strong synergy in all cell lines. The blocking of p-Y15-CDK1 marks the activation of CDK1/cyclin B, leading to premature entry into mitosis (pHH3) and accumulation of DNA damage (γH3AX), which could be observed in both sensitive and resistant cells with varying magnitude and kinetics (Fig. 4E). Significant downregulation of CHK1 was also detected in response to each drug and their combination, although this could be the consequence of an altered cell cycle. Collectively, blockade of AZD1775-induced feedback activation of CHK1 by SRA737 is likely the core mechanism driving the synergy between AZD1775 and SRA737 in prostate cancer cells.

### Inhibition of WEE1 or CHK1 suppressed tumor growth and metastasis in vivo.

**Figure 5:**
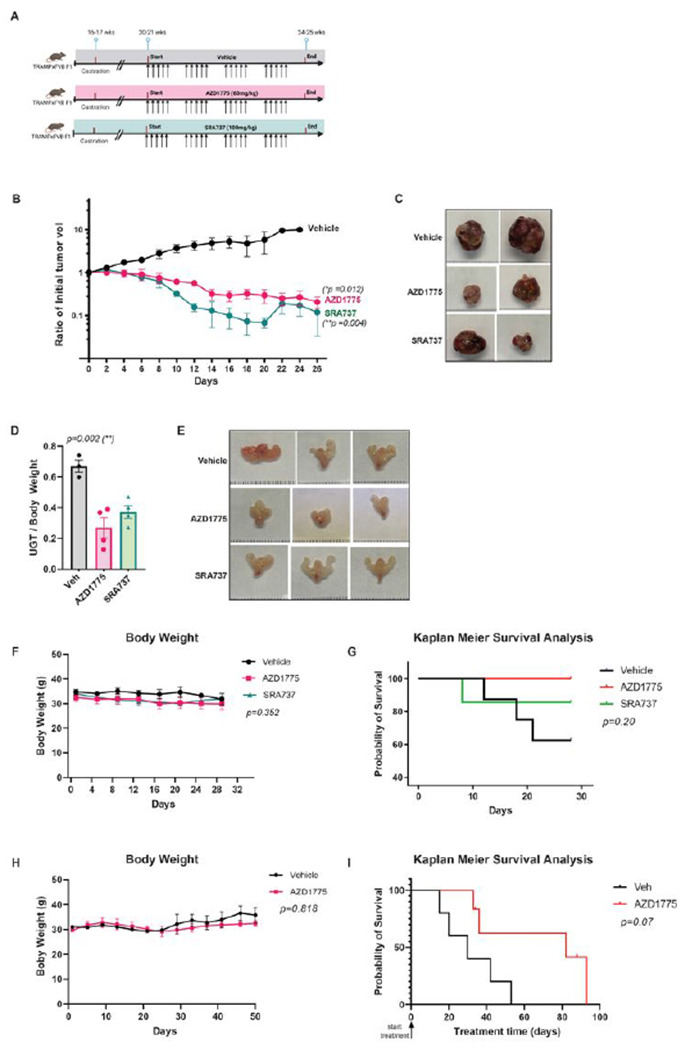
AZD1775 and SRA737 alone suppressed NEPC tumor growth in TRAMP mice. **A.** A diagram depicting the 4-week treatment plan. Castrated TRAMPxFVB F1 male mice were randomized to receive vehicle (n = 5), AZD1775 (n = 7), and SRA737 (n = 6) for 4 weeks. Created with BioRender.com. **B.**Tumor growth in mice with palpable tumors (***p < 0.001). **C.**Representative images of the dissected tumors. **D.** Final mean weight of urogenital tract (UGT) normalized to body weight in mice without palpable tumors. **E.** Representative image of UGTs in mice without palpable tumors. **F.** Mouse body weight over time. **G.** Kaplan-Meier survival analysis on vehicle- and drug-treated TRAMP mice (log-rank test). Statistical analysis by log-rank (Mantel-Cox) test. **H-I.**Mouse survival in response to AZD1775. Castrated TRAMP mice were randomized and treated with vehicle or AZD1775 (60 mg/kg, PO once daily) till humane endpoints. Body weight was monitored over time (H). Kaplan-Meier survival analysis on the vehicle- and AZD1775-treated TRAMP mice (log-rank test). Statistical significance (*p*values) is marked on each graph. The analysis method and data are shown in supplemental Table S3-S4.

To determine the therapeutic potential of AZD1775, SRA737, and their combination for treating advanced prostate cancer, we evaluated the efficacy *in vivo* using the castrated TRAMP mouse model, the best-known transgenic mouse model of NEPC. NEPC represents the most advanced stage of lethal prostate cancer, for which no effective therapies are available. TRAMP mice were routinely obtained as [TRAMPxFVB] F1 offspring. TRAMP tumors are characterized by rapid onset and metastatic progression with complete penetrance, and castration leads to the development of castration-resistant neuroendocrine carcinomas with lymph node and distal metastases [29]. We first evaluated the efficacy of AZD1775 and SRA737 as single agents in a 4-week simultaneous treatment regimen. TRAMP mice castrated at 16–17 weeks of age were randomized into three treatment groups: vehicle (n = 8), AZD1775 (60 mg/kg, n = 7), and SRA737 (100 mg/kg, n = 7) (Fig. 5A). All treatments were administered orally on 5 days-on / 2 days-off for four cycles. The mice were euthanized after four weeks. In mice with palpable tumors, tumor size was determined twice a week until the end of the experiment. Owing to the considerable variability in tumor volumes and stages, data from mice with or without palpable tumors were stratified and analyzed separately. In mice with palpable prostate tumors, AZD1775 or SRA737 administered alone not only completely blocked tumor growth but also resulted in tumor regression in both treatment groups (*p= 0.012 for AZD1775; **p = 0.004 for SRA737) (Fig. 5B–C and statistical analysis in **Supplemental Table S3**). In mice without palpable tumors, the final mean urogenital tract (UGT) weight from AZD1775- or SRA737-treated mice was significantly lower than that of vehicle-treated mice (**p < 0.002) (Fig. 5D and statistical analysis in **supplemental Table S4**). Correspondingly, the sizes of the UGTs were also smaller in drug-treated mice (Fig. 5E). Each drug was well tolerated because mouse body weight remained constant throughout the treatment (*p* = 0.352) and no other adverse effects were observed (Fig. 5F and statistical analysis in **Supplemental Table S4**). Kaplan–Meier survival analysis of mice treated with AZD1775 or SRA737 showed improved overall survival compared to the vehicle group, but the difference was not statistically significant (*p* = 0.20 with log-rank test) (Fig. 5G).

We demonstrated elevated WEE1 expression and potent cell-killing effects of AZD1775 in NEPC cells and tumor spheroids (Fig. 1), which is consistent with the evidence that WEE1 was previously identified as a potential target for NEPC tumors [6]. To fully exploit the therapeutic potential of WEE1 inhibition, we further examined the effectiveness of AZD1775 in a mouse survival experiment, in which treatment was initiated upon the development of palpable primary prostate tumors and continued until death or humane moribund endpoints. TRAMP male mice were castrated at 15–18 weeks. Upon development of palpable tumors, the mice were randomly assigned to two experimental groups: 1. vehicle (PO, qd, n = 5), 2. AZD1775 (60 mg/kg, PO, qd, n = 6). AZD1775 treatment for up to 12 weeks was well tolerated, with no significant change in body weight (*p* = 0.818) or apparent gross toxicity (Fig. 5H). Our data demonstrated that AZD1775 improved the overall survival of TRAMP mice compared to vehicle-treated mice, though did not reach statistical significance (*p* = 0.07 with log-rank test) (Fig. 5I).

In addition to tumor growth, the incidence of metastatic spread was examined visually at necropsy, and metastatic lesions were confirmed histologically. In this mouse model, lymph nodes were the most common sites of metastases, which occurred in 33%-40% of castrated mice at 24–25 weeks. As shown in Table 3, the incidence of lymph node (LN) metastases was lower in the AZD1775- and SRA737-treated groups than in the vehicle group. Metastases in other distal organs (liver, lung, and kidney) were also lower in drug-treated groups. Additionally, the incidence of newly developed primary prostate tumors during treatment was reduced in AZD1775- and SRA737-treated mice (Table 4). Taken together, the inhibition of WEE1 or CHK1 effectively suppresses the growth and metastasis of NEPC prostate tumors, supporting their values as single agents for prostate cancer treatment.

### Combined inhibition of WEE1 and CHK1 blocked NEPC tumor progression and metastasis.

**Figure 6:**
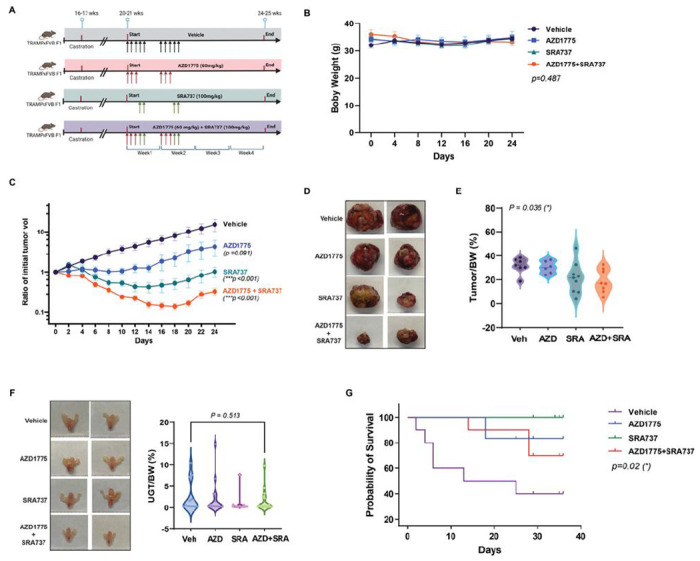
SRA737 and its combination with AZD1775 were more potent at suppressing tumor growth than AZD1775 in TRAMP mice. **A.** A diagram depicting the 2-week sequential treatment plan. Four groups of castrated TRAMPxFVB F1 male mice were randomized to receive vehicle (n = 20), AZD1775 (n = 20), and SRA737 (n = 23), and AZD1775+SRA737 (n = 25) following specified schedules and doses for 2 weeks. The mice were sacrificed after 4 weeks. Created with BioRender.com. **B.** Mouse body weight. **C.** Tumor growth in mice with palpable tumors (***p < 0.001). **D.** Representative images of the dissected tumors. **E.** Final tumor weight normalized to body weight in mice with palpable tumors. **F.**Final mean weight of urogenital tract (UGT) normalized to body weight in mice without palpable tumors. Representative images of UGTs are shown to the left. **G.** Kaplan–Meier survival analysis of mice with tumors. Statistical significance (p values) is marked on each graph. The analysis method and data are shown in supplemental Table S5-S6.

The effectiveness and tolerability of AZD1775 and SRA737 as single agents and their strong synergy in prostate cancer cells support their combination for increased efficacy and reduced toxicity. However, in a pilot experiment, a 4-week combination treatment showed signs of severe toxicity, including rapid weight loss and death. Careful optimization of the combination is required to maximize the efficacy and minimize toxicity. Therefore, we evaluated several combination regimens by varying the frequency, duration, and sequence of drug administration, and found that a sequential 2-week schedule was well tolerated. In a large scale study, four groups of randomized castrated TRAMP mice (20–21 weeks) were treated with vehicle (n = 20), AZD1775 (60 mg/kg, PO, 3 days on / 4 days off for 2 cycles, n = 20), SRA737 (100 mg/kg, PO, 2 days on / 5 days off for 2 cycles, n = 23), or AZD1775 (60 mg/kg) + SRA737 (100 mg/kg SRA737)(PO, 3 days AZD1775 followed by 2 days SRA737 / 2 days off for 2 cycles, n = 25) (Fig. 6A). Tumor growth in mice with palpable tumors was determined twice per week until the end of the experiment (Fig. 6C–D). Mice were euthanized after 4 weeks, and necropsy was performed to collect tissues and determine the extent of tumor development and incidence of tumor metastases. Sequential combination treatment was well tolerated. There was no significant difference in the body weight between the groups (*p* = 0.487) (Fig. 6B). No other gross toxicity was noted, and the internal organs (liver, spleen, and kidney) were normal in size/weight/appearance in all groups of mice (**Supplemental Fig. S4A**). In mice with palpable prostate tumors, the combination of AZD1775 and SRA737 provided the most potent suppression of tumor growth compared to each drug alone (*p* < 0.001). SRA737 also showed remarkable single-agent activity in tumor suppression (*p* < 0.001) compared to AZD1775, which was visible but not significant (*p* = 0.091) (Fig. 6C and statistical analysis in **Supplemental Table S5**). Accordingly, SRA737 and its combination also significantly reduced the final tumor weight (*p* < 0.05) (Fig. 6C–E and statistical analysis in **Supplemental Table S6**). After the cessation of drug treatment, tumor growth rebounded in the drug-treated groups. Nonetheless, a notable more sustained suppression of tumor growth (lasting until day 18) was observed in the combination-treated group (Fig. 6C). In mice without palpable tumors, the final mean %UGT weight was lower in the SRA737 (0.87 ± 1.98) and the combination groups (1.56 ± 2.49) as compared to vehicle (2.44 ± 3.54) and AZD1775 (2.32 ± 4.06). Still, the difference did not reach statistical significance (*p* = 0.513) (Fig. 6F). Kaplan–Meier survival analysis of mice treated with AZD1775 or SRA737 showed improved overall survival compared with the vehicle (*p* < 0.02) (Fig. 5G). The 28-day survival rates were 77% (vehicle), 91% (AZD), 100% (SRA), and 89% (AZD + SRA). Among the mice with tumors, the 28-day survival was 40% (vehicle), 83% (AZD), 100% (SRA), and 70% (AZD + SRA). Survival between these groups was better for all treatment groups than for the vehicle (*p* = 0.06 for all mice and *p* = 0.02 for mice with tumors only). Regarding tumor metastasis, the incidence of lymph node metastases at three sites (pelvic, renal, and inguinal LN) was lower in SRA737 and the combination groups than in the vehicle and AZD1775 groups. Metastasis in the distal organs (liver, lung, and kidney) was reduced in all treatment groups compared to that in the vehicle group. Notably, the effect of SRA737 on tumor metastasis was equivalent to or greater than that of the combination (Table 3). Additionally, the incidence of newly occurring primary prostate tumors was lowered in all treatment groups than in the vehicle group, with the most significant effect in the SRA737 group (Table 4). Collectively, these data demonstrated not only the synergistic effects of the AZD1775 and SRA737 combination, but also the effectiveness of SRA737 as a single agent in suppressing NEPC tumor growth and metastasis in castrated TRAMP mice.

## Discussion

Metastatic CRPC is a major clinical challenge, with few effective treatment options. This study investigated the therapeutic potential of targeting WEE1 and/or CHK1, two key G2/M and DDR regulators, for the treatment of CRPC *in vitro* and *in vivo*. Our data indicate that AZD1775, a WEE1 inhibitor, and SRA737, a CHK1 inhibitor, synergistically killed CRPC cells *in vitro*. We also demonstrated for the first time that both agents, alone or in combination, can block the progression and metastatic spread of CRPC tumors in a TRAMP prostate cancer mouse model. Notably, a 2-week sequential combination treatment with AZD1775 and SRA737 was more effective in suppressing tumor growth than each drug alone. SRA737, as a single agent, also exhibited remarkable efficacy in blocking CRPC tumor growth and metastasis and increasing survival in the TRAMP mouse model. These findings demonstrate the value of targeting WEE1 and CHK1 for CRPC treatment.

The frequent loss or mutation of *TP53*, *RB1*, and *PTEN* tumor suppressor genes in CRPC could make cancer cells dependent on the G2/M checkpoint for survival, increasing their sensitivity to G2/M checkpoint targeted agents [3]. Indeed, we observed upregulation of WEE1 and CHK1 expression in CRPC patient tumors and cell lines. Interestingly, increased WEE1 and CHK1 expression did not translate into higher sensitivity to their inhibitors. In addition, the difference in sensitivity to WEE1 and CHK1 inhibitors did not correlate with the mutation status of *TP53*, *RB1*, and *PTEN* in CRPC cell lines, suggesting more complex mechanisms underlying their sensitivity to these agents. Despite the differential sensitivity to SRA737, strong synergy between AZD1775 and SRA737 was demonstrated in all CRPC and NEPC cell lines and NEPC tumor spheroids, supporting the therapeutic value of this combination for CRPC. At the mechanistic level, our analysis indicated that the synergy between these two drugs centered on the blockade of WEE1 inhibition-induced feedback activation of CHK1 (measured by p-S345-CHK1) by the CHK1 inhibitor SRA737. In SRA737-resistant cells, despite a greater upregulation of p-S345-CHK1 induced by AZD1775, SRA737 was equally effective in blocking this feedback mechanism, resulting in nearly complete inhibition of p-Y15-CDK1 by the two-drug combination, which led to the activation of CDK1/cyclin B1, premature mitotic entry, and apoptotic cell death. Our findings agreed with another report on WEE1 and CHK1 inhibition combination in head and neck squamous cell carcinomas cells [30]. The synthetic lethal interactions of AZD1775 and SRA737 in CRPC cells provided a sound rationale for testing this combination in preclinical settings.

Combinations of WEE1 and CHK1 inhibitors have not been investigated in any prostate cancer model, although there were a few reports of this combination in other cancer models [30–34]. Our study is the first that examined this combination in an autochthonous genetically engineered transgenic mouse model of prostate cancer that provided valuable information on the efficacy of this combination in tumor metastasis along with tumor growth. Our study is also the first that evaluated the CHK1 inhibitor SRA737 in prostate cancer, and its combination with AZD1775 as this combination *per se* has not been assessed in any cancer models. Our study fills these knowledge gaps and provides a valuable preclinical assessment of this inhibitor combination for the treatment of lethal prostate cancers. We evaluated two combination regimens: a simultaneous 4-week schedule and a sequential 2-week schedule. Although substantial toxicity was associated with this combination in the simultaneous 4-week schedule, a shorter sequential treatment schedule was proven effective in maximizing efficacy and reducing toxicity. This sequential strategy was based on our finding that AZD1775 induced feedback activation of CHK1; thereby, administering the CHK1 inhibitor after the WEE1 inhibitor will eliminate this undesired effect. Corroborating this strategy, the sequential combination demonstrated synergy between the two drugs in suppressing tumor growth and metastasis. Notably, the efficacy of the combination in reducing incidence of metastatic spread was comparable with that of SRA737 alone, suggesting that inhibition of CHK1 playa predominant role in this context. These are important findings as tumor metastasis is the direct cause of high mortality for this disease. As tumors in TRAMP mice are considered NEPC- like, our study also has implications for treating NEPC, an end-stage aggressive CRPC that is AR-negative and resistant to AR-targeted therapies.

In our study, SRA737 exhibited significant single-agent activity in suppressing CRPC tumor growth and metastasis in a TRAMP mouse model. It was also well-tolerated in the 4-week treatment study, with no consequential body weight loss or gross toxicity. These preclinical data support the potential of SRA737 as a treatment for CRPC. CHK1 inhibitors have been investigated in many clinical and preclinical studies. Despite their promising therapeutic potential [12], the low selectivity and toxicity often associated with CHK1 inhibitors have been the primary roadblocks for translating these agents to the clinic [35]. SRA737 is a newer generation and highly selective orally available CHK1 inhibitor that exhibits > 1000-fold selectivity against CHK2 and CDK1 (IC50: 0.32 nM for CHK1 and 697 nM for CHK2) [36, 37]. SRA737 demonstrated favorable safety and efficacy when combined with gemcitabine in preclinical non-small cell lung cancer (NSCLC) and MYC-driven B-cell lymphoma mouse models [36]. Importantly, recent reports from the only two clinical trials on SRA737 in advanced cancers (NCT02797964 and NCT02797977) demonstrated unique features of this drug, including lower myelotoxicity and higher but manageable GI toxicities that are distinct from other CHK1 inhibitors examined in the clinic [21, 38]. Particularly, despite no complete or partial RECIST responses were observed in one trial that included 13 mCRPC patients, 8/13 (61.5%) mCRPC patients showed stable disease after four cycles of therapy, demonstrating its potential for use in combination therapy.

In summary, our study identified WEE1 and CHK1, two critical CCR and G2/M checkpoint regulators, as potential novel therapeutic targets for CRPC. We further demonstrated the efficacy and tolerability of the WEE1 inhibitor AZD1775 and CHK1 inhibitor SRA737 as single agents and in combination for CRPC/NEPC treatment in a transgenic mouse model of advanced prostate cancer. Our findings provide solid preclinical support for the further evaluation of these agents for the treatment of lethal prostate cancer.

## Figures and Tables

**Table 1:** IC_50_ values for AZD1775 and SRA737 in CRPC and NEPC cell lines.

	IC_50_ (μM)^A^	
Cell Lines	AZD1775 n = 3	SRA737 n = 3
LNCaP^B^	0.26 ± 0.04	2.01 ± 0.53
22Rv1	0.71 ± 0.19	1.70 ± 0.33
DU145	0.70 ± 0.10	1.47 ± 0.61
C4-2B-MDVR	0.57 ± 0.23	2.21 ± 0.68
C4-2B-TaxR	0.66 ± 0.30	1.97 ± 1.15
C4-2B-AbiR	0.77 ± 0.1 6	9.29 ± 5.72
C4-2B	0.58 ± 0.04	20.17 ± 5.24
PC3	1.21 ± 0.69	18.60 ± 6.26
NCI-H660^C^	1.14 ± 0.29	23.18 ± 1.08

A IC_50_s were calculated using time-lapse imaging analysis of NR-positive cells treated with increasing concentrations of AZD1775 and SRA737 at 96 h. Data are Mean ± SEM from three independent experiments.

B IC_50_s for LNCaP were obtained based on % confluency imaged under phase.

C IC_50_s for H660 were obtained from the CCK-8 cell viability assay.

**Table 2:** Synergy scores from AZD1775 and SRA737 cooperativity screen.

	Loewe		Bliss		ZIP		HSA	
Cell lines	Score	*p*	Score	*p*	Score	*p*	Score	*p*
22Rv1	6.18	3.40E-06	3.2	1.16E-01	2.56	1.81E-01	10.17	6.21E-06
DU145	7.12	1.37E-10	0.02	9.92E-01	−2.17	2.20E-01	11.33	3.02E-06
C4-2B	8.88	1.94E-04	20.05	7.81E-09	17.22	8.64E-08	15.39	7.84E-08
C4-2B-MDVR PC3	9.71	7.48E-10	7.33	1.35E-05	6.77	1.59E-05	12.97	1.26E-10
PC3	18.98	9.27E-09	32.43	1.96E-13	31.17	8.79E-12	25.13	4.89E-12
LASCPC-01	4.62	8.91E-07	9.78	6.88E-08	9.06	2.05E-06	13.97	9.46E-32

Cell viability (%) was calculated by Incucyte live cell imaging analysis. Drug interactions at day 4 were analyzed by SyngeryFinder Plus. Synergy scores and *p* values were obtained from four synergy analysis models. Representative data from one of three independent experiments are shown.

**Table 3:** Incidence of distant metastases confirmed histologically.

		Pelvic LN			Renal LN			Inguinal LN			All 3 LNs			Distal Organs		
Treatment	N^A^	+^B^	%	*p* ^D^	+^C^	%	*p*	+^C^	%	*p*	+^C^	%	*p*	+^C^	%	*p*
**4-wk treatment**				1			0.726			1			0.726			0.623
**Vehicle**	5	2	40		1	20		0	0		1	20		2	40	
**AZD1775**	7	2	29		1	14		1	14		1	14		1	14	
**SRA737**	6	2	33		0	0		1	17		0	0		1	17	
**2-wk sequential treatment**				0.305			1			0.084			0.648			0.207
**Vehicle**	20	11	55.0		5	25.0		4	20.0		1	5.0		3	15.0	
**AZD1775**	20	11	55.0		4	20.0		6	30.0		3	15.0		1	5.0	
**SRA737**	23	9	39.1		5	21.7		1	4.3		1	4.3		0	0	
**AZD1775 + SRA737**	25	8	32.0		6	24.0		2	8.0		2	8.0		1	4.0	

A Total number of mice in each experimental group, including all mice with or without palpable tumors at the beginning of the treatment. The numbers do not include mice that died during the treatment (3 died for vehicle and 1 for SRA737). LN: Lymph node.

B Number of mice with positive lymph node metastases in indicated lymph nodes. The presence of metastatic tumor cells in the lymph nodes was confirmed histologically by staining for synaptophysin.

C Number of mice with positive metastases in distal organs including urethra, liver, lung, or kidney. The presence of metastases was confirmed visually and histologically by staining for synaptophysin.

D *p* values are obtained from Fisher’s exact test (FET) evaluating the association between developing recurrence and treatment group. Computed FET across all groups (2×3 or 2×4 tables).

**Table 4:** Incidence of relapsed and newly occurred primary prostate tumors.

		Macroscopic recurred prostate tumor			Microscopic recurred prostate tumor		
Treatment	n^A^	n^B^	%	*p* ^D^	n^C^	%	*p*
**4-wk treatment**							0.726
**Vehicle**	5	2	40.0		1	20.0	
**AZD1775**	7	2	28.6		1	14.3	
**SRA737**	6	2	33.3		0	0	
**2-wk sequential treatment**				0.188			0.789
**Vehicle**	14	7	50.0		2	14.3	
**AZD1775**	14	5	35.7		1	7.14	
**SRA737**	15	2	13.3		1	6.67	
**AZD1775 + SRA737**	16	4	25.0		3	18.8	

A Total number of mice in each experimental group including all mice without tumors at the beginning of treatment. The numbers do not include mice that died during the treatment (3 died for vehicle and 1 for SRA737).

B Number of mice with palpable (macroscopic) prostate tumors recurred during drug treatment.

C Number of mice with microscopic prostate tumors recurred during drug treatment. The presence of primary tumors was confirmed histologically by H&E and staining for synaptophysin.

D *p* values were obtained from Fisher’s exact test (FET) evaluating the association between developing recurrence and treatment group. Computed FET across all groups (2X3 or 2X4 tables).

## Data Availability

The data generated in this study are available in the article and its supplementary files. Raw and derived data supporting the findings of this study, but not listed in detail, are available from the corresponding author upon request. Expression profile data analyzed in this study were obtained from the Cancer Genome Atlas, including the NEPC dataset [20] (all accessible via https://portal.gdc.cancer.gov/), and publicly available data from the SU2C/PCF Dream Team [15].

## Abbreviations

CHK1: CHEK1
CRPC: castration-resistant prostate cancer
mCRPC: metastatic castration-resistant prostate cancer
NEPC: neuroendocrine prostate cancer
tNEPC: treatment-related neuroendocrine prostate cancer
ADT: androgen deprivation therapy
TRAMP: Transgenic Adenocarcinoma of Mouse Prostate
AZD: AZD1775
SRA: SRA737
IHC: immunohistochemistry
IF: immunofluorescence
pHH3: p-S10-Histone H3
γH2AX: p-S139-His H2A.X
WB: Western blotting
NR: NucLight Red
UGT: urogenital tract
LN: lymph node

## References

[1] Elbaek CR, Petrosius V, Sorensen CS. WEE1 kinase limits CDK activities to safeguard DNA replication and mitotic entry. Mutat Res 2020; 819-820: 111694. 32120135 10.1016/j.mrfmmm.2020.111694

[2] Qiu Z, Oleinick NL, Zhang J. ATR/CHK1 inhibitors and cancer therapy. Radiother Oncol 2018; 126: 450–464. 29054375 10.1016/j.radonc.2017.09.043 PMC5856582

[3] Visconti R, Della Monica R, Grieco D. Cell cycle checkpoint in cancer: a therapeutically targetable double-edged sword. J Exp Clin Cancer Res 2016; 35: 153. 27670139 10.1186/s13046-016-0433-9 PMC5037895

[4] Ghelli Luserna di Rora A, Cerchione C, Martinelli G, Simonetti G. A WEE1 family business: regulation of mitosis, cancer progression, and therapeutic target. J Hematol Oncol 2020; 13: 126. 32958072 10.1186/s13045-020-00959-2 PMC7507691

[5] Mak JP, Man WY, Chow JP, Ma HT, Poon RY. Pharmacological inactivation of CHK1 and WEE1 induces mitotic catastrophe in nasopharyngeal carcinoma cells. Oncotarget 2015; 6: 21074–21084. 26025928 10.18632/oncotarget.4020 PMC4673251

[6] Corella AN, Cabiliza Ordonio MVA, Coleman I, Lucas JM, Kaipainen A, Nguyen HM Identification of Therapeutic Vulnerabilities in Small-cell Neuroendocrine Prostate Cancer. Clinical cancer research : an official journal of the American Association for Cancer Research 2020; 26: 1667–1677. 31806643 10.1158/1078-0432.CCR-19-0775 PMC7124974

[7] Ku BM, Bae YH, Koh J, Sun JM, Lee SH, Ahn JS Mutational status of TP53 defines the efficacy of Wee1 inhibitor AZD1775 in KRAS-mutant non-small cell lung cancer. Oncotarget 2017; 8: 67526–67537. 28978051 10.18632/oncotarget.18728 PMC5620191

[8] Hirai H, Iwasawa Y, Okada M, Arai T, Nishibata T, Kobayashi M Small-molecule inhibition of Wee1 kinase by MK-1775 selectively sensitizes p53-deficient tumor cells to DNA-damaging agents. Mol Cancer Ther 2009; 8: 2992–3000. 19887545 10.1158/1535-7163.MCT-09-0463

[9] Kolb EA, Houghton PJ, Kurmasheva RT, Mosse YP, Maris JM, Erickson SW Preclinical evaluation of the combination of AZD1775 and irinotecan against selected pediatric solid tumors: A Pediatric Preclinical Testing Consortium report. Pediatr Blood Cancer 2020; 67: e28098. 31975571 10.1002/pbc.28098 PMC8752046

[10] Richer AL, Cala JM, O’Brien K, Carson VM, Inge LJ, Whitsett TG. WEE1 Kinase Inhibitor AZD1775 Has Preclinical Efficacy in LKB1-Deficient Non-Small Cell Lung Cancer. Cancer Res 2017; 77: 4663–4672. 28652249 10.1158/0008-5472.CAN-16-3565

[11] Matheson CJ, Venkataraman S, Amani V, Harris PS, Backos DS, Donson AM A WEE1 Inhibitor Analog of AZD1775 Maintains Synergy with Cisplatin and Demonstrates Reduced Single-Agent Cytotoxicity in Medulloblastoma Cells. ACS Chem Biol 2016; 11: 921–930. 26745241 10.1021/acschembio.5b00725

[12] Patties I, Kallendrusch S, Bohme L, Kendzia E, Oppermann H, Gaunitz F The Chk1 inhibitor SAR-020106 sensitizes human glioblastoma cells to irradiation, to temozolomide, and to decitabine treatment. J Exp Clin Cancer Res 2019; 38: 420. 31639020 10.1186/s13046-019-1434-2 PMC6805470

[13] Doerr F, George J, Schmitt A, Beleggia F, Rehkamper T, Hermann S Targeting a non-oncogene addiction to the ATR/CHK1 axis for the treatment of small cell lung cancer. Sci Rep 2017; 7: 15511. 29138515 10.1038/s41598-017-15840-5 PMC5686113

[14] Nyquist MD, Corella A, Coleman I, De Sarkar N, Kaipainen A, Ha G Combined TP53 and RB1 Loss Promotes Prostate Cancer Resistance to a Spectrum of Therapeutics and Confers Vulnerability to Replication Stress. Cell Rep 2020; 31: 107669. 32460015 10.1016/j.celrep.2020.107669 PMC7453577

[15] Abida W, Cyrta J, Heller G, Prandi D, Armenia J, Coleman I Genomic correlates of clinical outcome in advanced prostate cancer. Proc Natl Acad Sci U S A 2019; 116: 11428–11436. 31061129 10.1073/pnas.1902651116 PMC6561293

[16] Heagerty PJ, Lumley T, Pepe MS. Time-dependent ROC curves for censored survival data and a diagnostic marker. Biometrics 2000; 56: 337–344. 10877287 10.1111/j.0006-341x.2000.00337.x

[17] Liu Q, Yin X, Languino LR, Altieri DC. Evaluation of drug combination effect using a Bliss independence dose-response surface model. Stat Biopharm Res 2018; 10: 112–122. 30881603 10.1080/19466315.2018.1437071 PMC6415926

[18] Gingrich JR, Barrios RJ, Morton RA, Boyce BF, DeMayo FJ, Finegold MJ Metastatic prostate cancer in a transgenic mouse. Cancer Res 1996; 56: 4096–4102. 8797572

[19] Roy A, Veroli MV, Prasad S, Wang QJ. Protein Kinase D2 Modulates Cell Cycle By Stabilizing Aurora A Kinase at Centrosomes. Mol Cancer Res 2018; 16: 1785–1797. 30018032 10.1158/1541-7786.MCR-18-0641 PMC9923726

[20] Beltran H, Prandi D, Mosquera JM, Benelli M, Puca L, Cyrta J Divergent clonal evolution of castration-resistant neuroendocrine prostate cancer. Nat Med 2016; 22: 298–305. 26855148 10.1038/nm.4045 PMC4777652

[21] Kristeleit R, Plummer R, Jones R, Carter L, Blagden S, Sarker D A Phase 1/2 trial of SRA737 (a Chk1 inhibitor) administered orally in patients with advanced cancer. Br J Cancer 2023; 129: 38–45. 37120671 10.1038/s41416-023-02279-x PMC10307885

[22] Gorecki L, Andrs M, Korabecny J. Clinical Candidates Targeting the ATR-CHK1-WEE1 Axis in Cancer. Cancers (Basel) 2021; 13. 33672884 10.3390/cancers13040795 PMC7918546

[23] Booth L, Roberts J, Poklepovic A, Dent P. The CHK1 inhibitor SRA737 synergizes with PARP1 inhibitors to kill carcinoma cells. Cancer Biol Ther 2018; 19: 786–796. 30024813 10.1080/15384047.2018.1472189 PMC6154848

[24] Zhu Y, Liu C, Nadiminty N, Lou W, Tummala R, Evans CP Inhibition of ABCB1 expression overcomes acquired docetaxel resistance in prostate cancer. Mol Cancer Ther 2013; 12: 1829–1836. 23861346 10.1158/1535-7163.MCT-13-0208 PMC3947549

[25] Liu C, Lou W, Zhu Y, Nadiminty N, Schwartz CT, Evans CP Niclosamide inhibits androgen receptor variants expression and overcomes enzalutamide resistance in castration-resistant prostate cancer. Clinical cancer research : an official journal of the American Association for Cancer Research 2014; 20: 3198–3210. 24740322 10.1158/1078-0432.CCR-13-3296 PMC4058390

[26] Liu C, Lou W, Zhu Y, Yang JC, Nadiminty N, Gaikwad NW Intracrine Androgens and AKR1C3 Activation Confer Resistance to Enzalutamide in Prostate Cancer. Cancer Res 2015; 75: 1413–1422. 25649766 10.1158/0008-5472.CAN-14-3080 PMC4383695

[27] Leung-Pineda V, Ryan CE, Piwnica-Worms H. Phosphorylation of Chk1 by ATR is antagonized by a Chk1-regulated protein phosphatase 2A circuit. Mol Cell Biol 2006; 26: 7529–7538. 17015476 10.1128/MCB.00447-06 PMC1636880

[28] Bukhari AB, Lewis CW, Pearce JJ, Luong D, Chan GK, Gamper AM. Inhibiting Wee1 and ATR kinases produces tumor-selective synthetic lethality and suppresses metastasis. J Clin Invest 2019; 129: 1329–1344. 30645202 10.1172/JCI122622 PMC6391092

[29] Gingrich JR, Barrios RJ, Kattan MW, Nahm HS, Finegold MJ, Greenberg NM. Androgen-independent prostate cancer progression in the TRAMP model. Cancer Res 1997; 57: 4687–4691. 9354422

[30] Deneka AY, Einarson MB, Bennett J, Nikonova AS, Elmekawy M, Zhou Y Synthetic Lethal Targeting of Mitotic Checkpoints in HPV-Negative Head and Neck Cancer. Cancers (Basel) 2020; 12. 32012873 10.3390/cancers12020306 PMC7072436

[31] Carrassa L, Chila R, Lupi M, Ricci F, Celenza C, Mazzoletti M Combined inhibition of Chk1 and Wee1: in vitro synergistic effect translates to tumor growth inhibition in vivo. Cell Cycle 2012; 11: 2507–2517. 22713237 10.4161/cc.20899

[32] Magnussen GI, Emilsen E, Giller Fleten K, Engesaeter B, Nahse-Kumpf V, Fjaer R Combined inhibition of the cell cycle related proteins Wee1 and Chk1/2 induces synergistic anti-cancer effect in melanoma. BMC Cancer 2015; 15: 462. 26054341 10.1186/s12885-015-1474-8 PMC4460948

[33] Koh SB, Wallez Y, Dunlop CR, Bernaldo de Quiros Fernandez S, Bapiro TE, Richards FM Mechanistic Distinctions between CHK1 and WEE1 Inhibition Guide the Scheduling of Triple Therapy with Gemcitabine. Cancer Res 2018; 78: 3054–3066. 29735549 10.1158/0008-5472.CAN-17-3932 PMC5985963

[34] Guertin AD, Martin MM, Roberts B, Hurd M, Qu X, Miselis NR Unique functions of CHK1 and WEE1 underlie synergistic anti-tumor activity upon pharmacologic inhibition. Cancer Cell Int 2012; 12: 45. 23148684 10.1186/1475-2867-12-45 PMC3517755

[35] Sausville E, Lorusso P, Carducci M, Carter J, Quinn MF, Malburg L Phase I dose-escalation study of AZD7762, a checkpoint kinase inhibitor, in combination with gemcitabine in US patients with advanced solid tumors. Cancer Chemother Pharmacol 2014; 73: 539–549. 24448638 10.1007/s00280-014-2380-5 PMC4486055

[36] Walton MI, Eve PD, Hayes A, Henley AT, Valenti MR, De Haven Brandon AK The clinical development candidate CCT245737 is an orally active CHK1 inhibitor with preclinical activity in RAS mutant NSCLC and Emicro-MYC driven B-cell lymphoma. Oncotarget 2016; 7: 2329–2342. 26295308 10.18632/oncotarget.4919 PMC4823038

[37] Osborne JD, Matthews TP, McHardy T, Proisy N, Cheung KM, Lainchbury M Multiparameter Lead Optimization to Give an Oral Checkpoint Kinase 1 (CHK1) Inhibitor Clinical Candidate: (R)-5-((4-((Morpholin-2-ylmethyl)amino)-5-(trifluoromethyl)pyridin-2-yl)amino)pyr azine-2-carbonitrile (CCT245737). J Med Chem 2016; 59: 5221–5237. 27167172 10.1021/acs.jmedchem.5b01938

[38] Jones R, Plummer R, Moreno V, Carter L, Roda D, Garralda E A Phase I/II Trial of Oral SRA737 (a Chk1 Inhibitor) Given in Combination with Low-Dose Gemcitabine in Patients with Advanced Cancer. Clinical cancer research : an official journal of the American Association for Cancer Research 2023; 29: 331–340. 36378548 10.1158/1078-0432.CCR-22-2074 PMC10539020

